# Teacher Nomination of School-aged Children for Mental Health Services in a Low and Middle Income Country

**DOI:** 10.1080/16549716.2020.1861921

**Published:** 2021-02-16

**Authors:** Christina M. Cruz, Molly M. Lamb, Karen Hampanda, Priscilla Giri, Matthew Campbell, Bijita Chowdhury, Aileen A. Giardina, Bradley N. Gaynes, Michael Matergia

**Affiliations:** a Department of Psychiatry, University of North Carolina at Chapel Hill School of Medicine, Chapel Hill, NC, USA; b Department of Epidemiology, Colorado School of Public Health, Aurora, CO, USA; c Center for Global Health, Colorado School of Public Health, Aurora, CO, USA; d Department of Child Mental Health, Darjeeling Ladenla Road Prerna, Darjeeling, India; e Broadleaf Health and Education Alliance, Stroudsburg, PA, USA; f Department of Family Medicine, St. Joseph Hospital, SCL Health, Denver, CO, USA

**Keywords:** Global mental health, child mental health, teacher identification, task-shifting, India

## Abstract

**Background**: Knowledgeable in child development, primary school teachers in low- and middle-income countries (LMICs) have the potential to identify their students needing mental health care.

**Objective**: We evaluated whether teachers in Darjeeling, India can accurately nominate school-aged children for mental health services after training and aided by a novel tool.

**Methods**: In 2018, 19 primary school teachers from five low-cost private (LCP) schools in rural Darjeeling were trained to nominate children needing care. Teachers evaluated all of their students aided by a novel tool, ‘Behavior Type and Severity Tool’ (BTST), completed the Achenbach Teacher Report Form (TRF) as a mental health status reference standard, and nominated two students for care. Sensitivity and specificity of being nominated compared to TRF overall and subdomain scores were calculated. BTST performance was determined by comparing BTST and TRF scores and creating Receiver Operating Characteristic curves to determine optimal cutoffs. Multivariable regression models were used to identify demographic predictors of teacher accuracy using the BTST.

**Results**: For students demonstrating a clinical or borderline score in at least one TRF subdomain, the sensitivity (72%) and specificity (62%) of teacher nomination were moderately high. BTST overall scores and TRF Total Problem scores were correlated (Spearman’s ρ = 0.34, p < 0.0001), as were all subdomains. For the TRF Total Problem score, a maximum Youden’s J of 0.39 occurred at BTST cutoff >4 for borderline struggles and 0.54 at the BTST cutoff >6 for clinical struggles. Younger teacher age, less education, less formal education training, and more years of experience were positively associated with teacher accuracy.

**Conclusions**: With training and a simple decision support tool, primary school teachers in an LMIC nominated students for mental health services with moderate accuracy. With the BTST being weakly accurate, teachers’ judgment largely accounted for the moderate accuracy of nominations.

## Background

Expanding access to children’s mental health care is critical. Worldwide prevalence of mental health conditions in children is reported at 10–20% [1,2]. Fewer than 1% of children who need mental health services in India, the low- and middle-income country (LMICs) study setting, are receiving care, a gap even larger than in high-income countries (HICs) where 20% of children needing mental health services receive care [1,3,4].

Referring children for mental health services is the first essential step to increase care access [1,5,6]. Given the scarcity in LMICs of mental health professionals and referral pathways, alternative pathways to identify and refer children are necessary to improve access [7]. In HICs, teachers have been well-studied alternative referrers of children, stemming from their natural role as consistent, attentive adults who observe children’s strengths and difficulties for many hours a day [8–10]. Moreover, teachers may have access to an overwhelming majority of school-aged children; the gross enrollment ratio at the primary school level in India, for instance, is estimated at 95%, though enrollment rates for those with mental health struggles have not been well documented [11,12].

In HICs, three teacher nomination methods have been studied: simple nomination, in-service, and standardized measures [13]. In simple nomination, teachers nominate their students for mental health services based on their professional experience and judgment [10]. Using simple nomination, teachers fairly accurately nominate children with externalizing behaviors and severe internalizing behaviors but overlook children with subclinical to moderate internalizing behaviors [8,10,14–18]. In-service involves training teachers to nominate students, typically focusing on one specific diagnosis; this training improves teachers’ ability to accurately nominate students compared to simple nomination [13,19]. Standardized measures that capture teacher input, the gold standard, have been shown to accurately capture clinical levels of student struggles in HICs [13,19].

HIC teacher nomination findings may not translate into LMIC contexts. With potential HIC-LMIC differences in teacher training content, duration, or rigor, simple nomination may be less accurate in LMICs [10,14,20]. Though considered a gold standard, use of standardized measures may not be sustainable in LMIC settings given the measures’ licensing fees, teacher time needed to administer the often lengthy measures, and the professional staff typically required to score and interpret the measures [13,19]. Burgeoning evidence, however, suggests that in-service teacher training in LMICs facilitates fairly accurate teacher nomination of youth for mental health services, though less accurate than standardized measures [6,21–26]. Complementing in-service with a brief, simple tool approximating standardized measures may further facilitate accurate teacher nomination in LMICs, but no published studies have taken this approach [6,13,22].

LMIC teacher nomination of students for mental health services after in-service training has been studied for the pre-school-age population, for whom many mental health concerns will not yet be evident, and for adolescents, for whom mental health struggles resemble those of adults and are considered easier to identify than in school-aged children [6,21–25]. Nominating school-aged children for mental health services is crucial as they are often overlooked for care despite being at the age when the potential for life-altering prevention of poor outcomes is highest [25]. Research on the in-service nomination of school-aged children has studied diagnosis-specific nomination [27,28] or identification of symptoms in theoretical examples, such as in case vignettes [29–31]. To our knowledge, no published studies have examined the ability of teachers in LMICs to broadly recognize mental health struggles in their school-aged students after in-service training [6,32].

Here, we studied whether primary school teachers could accurately nominate their students for mental health services after training and with the aid of a novel decision support tool. This analysis was a secondary aim of a study evaluating the feasibility of a task-shifting intervention in which teachers deliver evidence-based mental health services to their school-aged students whom they chose to receive services [33]. We created a simplified decision support tool, the ‘Behavior Type and Severity Tool’ (BTST) to help teachers systematically capture their mental health formulation of each student as one aspect to consider in their student nomination. In five rural, low-cost private (LCP) primary schools in the Darjeeling Himalayas of India, we compared teachers’ nominations of school-aged students for mental health services after training and aided by the BTST with the results of a validated measure for teacher assessment of children’s mental health, the Achenbach System of Empirically Based Assessment (ASEBA) Teacher Report Form (TRF) [34]. We hypothesized that, with in-service training and a simplified decision support tool, teachers can accurately nominate school-aged children in their classrooms for mental health services. We also evaluated the performance, validity, diagnostic accuracy, and alignment of the BTST relative to the gold-standard TRF. Finally, we explored whether any demographic characteristics of teachers or students were correlated with nomination accuracy.

## Methods

### Participants

The study occurred in rural, LCP schools of the Darjeeling Himalayas, West Bengal, India. The majority of the 800,000 person population is minority ethnic Nepali and lives in rural villages throughout the mountains [35]. The majority of workers (77%) earn daily wages of 120 INR (approximately 1.61 USD) [36]. Estimated at 30–50%, a growing population of children in Darjeeling are attending LCP schools that receive minimal government support and oversight [37–39]. No Darjeeling specific prevalence of child mental illness is available. In a nearby rural area in West Bengal, psychiatric morbidity among rural primary school children using the Rutter-B-Scale was estimated at 33.3%, though the Rutter-B-Scale was later found to have low sensitivity and specificity in India [2,40]. Conduct disorder was most prevalent, followed by enuresis, intellectual delay, simple disturbance of activity and attention, relationship problems, and hyperkinetic conduct disorder [2].

LCP schools in rural Darjeeling were eligible if they did not receive government aid, served students from families with an average daily income of 10 USD or less, and had annual fees totaling less than 180 USD. The task-shifting intervention was jointly pursued for research and community development, with a goal of reaching children with the poorest care access. To meet this goal, LCP criteria were based on a study in Darjeeling targeting children with poorer access to pediatric care [37]. Eligible schools were approached for participation in a year-long feasibility study of the task-shifting intervention. Seventeen schools were contacted, 11 agreed to participate, and 5 schools were chosen based on meeting inclusion criteria of having 50 or more students enrolled.

Teachers within enrolled schools were eligible if they had taught for at least 1 year, were 18 years or older, and were not suspected or convicted of child maltreatment. These criteria were chosen to maintain child safety and minimize confounding any findings with a teacher’s experience in classroom management. Twenty-three teachers were consented, of which 21 attended the 10-day training course for the full task-shifting intervention (part of which was dedicated to teacher identification of students needing mental health support, described in ‘Procedures’), and 19 completed the full activities of the study. Ethics approvals and consent procedures are detailed in ‘Ethics and consents’.

Once teachers were consented, local study representatives individually met with each student and family enrolled in the respective teacher’s class. 274 parents were approached for consent for their children’s participation in the study. 272 parents consented. Two parents refused participation. 272 students were assessed by their teachers and 36 (13%) were nominated for the mental health intervention based on a pragmatic limitation of two student nominations per teacher.

Teachers’ nominations were pragmatically limited as they were an entrée for children into the teacher-delivered task-shifting system of care. During intervention development, teachers expressed that delivering task-shifted mental health care their first time to two students was the maximum manageable workload. As the access to care is poor in Darjeeling, the nomination of more than two students per teacher would have resulted in these additional students not receiving care. Further, based on average class sizes of 14 students, choosing 2 students per class approximated the worldwide prevalence of childhood mental illness at 10–20% (though fell below local estimated prevalence) [1,2].

### Measures

#### Teacher Report Form (TRF)

Considered a gold standard, the TRF is a standardized measure filled out by teachers that maps teacher impressions of a student’s mental health concerns onto validated categories of mental health challenges in children [34]. It is typically scored and interpreted by those with mental health training. Clinical scores include a Total Problem score and several subdomains: dichotomous dimensions of Internalizing and Externalizing Problems, eight empirically validated syndromes (Aggressive Behavior, Rule-Breaking Behavior, Withdrawn/Depressed, Somatic Complaints, Anxious/Depressed, Attention Problems, Social Problems, and Thought Problems), and Diagnostic and Statistical Manual (DSM)-oriented subdomains [41]. Based on TRF author standards, students were characterized as normal, borderline, or clinical for each dimension and the Total Problem score. Raw scores obtained from these data were summed and converted into T-scores. Children with Total Problem t-scores lower than 60 are classified as ‘normal’, children with scores ranging from 60 to 63 are classified as ‘borderline’ and children with scores above 63 are classified as ‘clinical.’ Subdomain T-scores less than 65 are classified as ‘normal’, 65 to 69 classified as ‘borderline’, and 70 or greater classified as ‘clinical’. The form is available in Nepali and takes 20 minutes to complete.

The TRF has robust psychometric validity across various LMIC populations [42]. There is evidence for partial support for the factorial validity of the TRF in the Indian context [43,44]. Though lower TRF cutoff scores for ‘clinical’ and ‘borderline’ classifications were thought to be more appropriate for the Indian context, no other teacher report form reviewed by a panel of experts was as strongly validated for India or felt to be as locally relevant in Darjeeling as the TRF [43,44]. For instance, as previously mentioned, the Rutter-B-Scale used to evaluate children in rural West Bengal was later found to have low sensitivity (51.8%) and specificity (34.1%) in India [2,40]. To ensure accurate scoring, we applied the ASEBA Group 2 multi-cultural scoring framework [45].

#### Behavior Type and Severity Tool (BTST)

The BTST is a study-specific simple decision support tool (Supplemental Figure 1). Modeled after teacher nomination forms in HICs [10,46], this tool was developed to help teachers capture their clinical impressions of each student, a common mental health skill that teachers have less experience with [10,14]. Teachers assign each child an overall rating on a likert scale from 1 to 9 based on the teacher’s opinion (1–3: ‘does not need support’, 4–6: ‘might need support’, 7–9: ‘definitely needs support’). Teachers then identified (yes/no) if the student displayed one of the three behavior types they were trained on (anxious, disagreeable, or withdrawn) and could indicate ‘yes’ to more than one behavior category. The BTST was available in Nepali and took the teacher between 1 and 2 minutes to complete per student.

During the piloting of the BTST, we set a cut-off of 7–9 for teachers to designate the top third of children whom they felt ‘definitely needs support’, i.e., the approximately 10–33% of students expected to have child psychiatric morbidity in accordance with the literature [1–4]. We back-validated this cut-off after real-world application. The cut-off yielded an identification rate of 26% of students whom teachers felt ‘definitely needs support.’

BTST behavior categories reflected child mental health epidemiology literature that regularly grouped common diagnoses into behavior types of anxious, disruptive, or mood; the literature also suggested that diagnoses not typically fitting into these categories, such as psychosis or Autism, would still likely exhibit symptoms from one of these categories [47–51]. Category names of ‘anxious’, ‘disagreeable’, and ‘withdrawn’ were chosen after consulting with local experts and to avoid contributing to mental health stigma.

### Procedures

We used a pilot pragmatic design. Teacher nomination is one part of a novel task-shifting model in which the delivery of basic children’s mental health care is shifted to primary classroom teachers at LCP schools (Supplemental Figure 2) [33]. The feasibility study of the intervention, of which teacher nomination was one part, ran from January 2018 to December 2018. The trial was registered on 22/01/2018 with Clinical Trials Registry – India (CTRI), reg. no. CTRI/2018/01/011471, ref. no. REF/2017/11/015895.

A timeline of relevant procedures is outlined in Supplemental Figure 3. Teachers received a combined 12 hours of training to identify students with mental health concerns aided by the BTST. These hours were interspersed throughout a 10-day in-service training teaching teachers how to deliver task-shifted children’s mental health care (Supplemental Figure 4). Teachers learned to identify and understand the behaviors of children with mental health struggles through complex case vignettes. During training, they were provided with a workbook to guide their learning and study-specific tools (such as checklists) to reference when later delivering task-shifted care. Other than the BTST, they were not provided with study-specific tools to reference in identifying children in need of mental health support. The training was led by a psychiatric social worker with child mental health training and experience in delivering and supervising the delivery of task-shifted child mental health care.

After training, teachers observed their students for atypical behaviors for 3 months. In one visit, teachers completed the following procedures. Demographic information was collected on all consented children (n = 272). Teachers then formally evaluated their students’ need for mental health intervention (an average of 14 students per teacher) by completing the BTST for each child. Teachers thereafter filled out the TRF for each student, with study staff scoring TRFs after teacher nominations were completed. Without knowing students’ TRF scores, each teacher was asked to nominate two students and one alternate, based on their judgment, aided by their BTST ratings.

Several procedures were utilized to ensure the integrity of teachers’ assessments. During training, frequent checks for understanding (probing questions, self-assessment, and tasks) and a summative evaluation were used to assess comprehension. Following training, teachers received monthly in-person supervision and monthly phone supervision. Study staff were present during each teacher’s nomination process to answer questions and promote accurate form completion.

### Data analysis

Demographics of the children that were nominated and not nominated were compared using the independent sample *t* test for continuous variables, χ^2^ for categorical variables, or Spearman’s Rank Correlation for ordinal variables. Sensitivity and specificity of a child’s nomination status (nominated or not, as aided by the BTST) as compared to having any positive TRF score were calculated. ‘Any positive TRF score’ was defined as having either a borderline or clinical TRF score on the Total Problem score or any subdomain. Sensitivity is interpreted as ‘what percent of children that were nominated had at least 1 clinical or borderline total or subdomain TRF score?’ while specificity is interpreted as ‘what percent of children that were not nominated had all normal total and subdomain TRF scores?’ Sensitivity and specificity were calculated in this way to account for the pragmatic limitation of two student nominations per teacher.

To examine the performance and validity of the BTST, we calculated the Spearman’s *rho* coefficient between the BTST and TRF for total and sub-domain scores. We mapped the TRF subdomains to the 3 BTST categories (anxious, withdrawn, disagreeable). The BTST categories were aligned with the TRF after a review of the child psychiatric epidemiology literature (Supplemental Table 1) [47–51].

To determine overall BTST diagnostic accuracy and if the BTST cutoffs chosen were in alignment with the TRF clinical and borderline definitions, we created three Receiver Operating Characteristics (ROC) curves. The first ROC curve compared increasing BTST cutoffs in children who had ‘clinical’ or ‘borderline’ TRF Total Problem scores versus children who had ‘normal’ TRF Total Problem scores; the second ROC curve compared increasing BTST cutoffs in children who had ‘clinical’ TRF Total Problem scores versus children who had ‘borderline’ or ‘normal’ TRF Total Problem scores; and the third ROC curve compared increasing BTST cutoffs in children who had any positive TRF score versus children who had all ‘normal’ TRF scores. The area under the curve (AUC) was calculated for each ROC curve to evaluate diagnostic accuracy. The Youden Index was calculated to determine the BTST score on each ROC curve at which BTST performance was maximized.

We conducted a multivariable generalized linear regression analysis on the 258 students for whom we had both BTST and TRF scores to determine which teacher demographic characteristics were associated with higher teacher accuracy (i.e. higher spearman’s *rho* coefficient between the BTST overall score and the TRF total problem T-score). We then explored the degree to which teacher demographic characteristics associated with higher teacher accuracy explained BTST score variability. We conducted a multilevel, multivariable generalized linear regression analysis to investigate, at the teacher level, the association of BTST scores (outcome) with TRF scores and with the teacher demographic characteristics associated with higher BTST – TRF rho scores. A multilevel analysis was conducted to directly account for individual teachers assessing multiple students and the corresponding variation in scores at the level of the individual teacher. We also conducted a multivariable generalized linear regression analysis to identify student demographic predictors of being nominated (yes/no) for mental health intervention by their classroom teacher. For the non-multilevel multivariable regression analyses, potentially predictive factors that showed a relatively strong association with the outcome in univariate regression analyses (p < 0.2) were tested for association with the outcome in multivariable generalized linear regression models. Backwards intentional elimination was used to determine which variables were independently, significantly associated with the outcome. Predictive variables retained in the final model were independently associated with the outcome at the p < 0.05 level. SAS version 9.4 (Cary, NC) was used for all data analysis [52].

## Results

Teachers who participated in the training were 79% female. 37% belonged to a scheduled caste or tribe. All teachers had at least higher secondary education, and most had undergraduate training; however, very few had formal training in education or a teaching certificate (Table 1). Enrolled students were approximately 50% female and evenly distributed amongst grades 1–4. The demographic characteristics of students across nomination status were similar, with the exception of age and age-grade mismatch (Table 2).

**Table 1.** Teacher demographics

Categorical variables	*n*		*%*
Gender Female Male	15 4		79.0 21.0
Scheduled caste/tribe^a^ Yes No	7 12		36.8 63.2
Language Nepali Bengali English Hindi Other	18 0 18 12 0		94.7 0 94.7 63.2 0
Formal training in education Yes No	4 15		21.0 79.0
Teaching certificate Yes No	3 16		15.8 84.2
Class levels taught^b^ Class I (kindergarten) Class II (1st grade) Class III (2nd grade) Class IV (3rd grade) Class V (4th grade) Class VI (5th grade) Class VII (6th grade)	9 14 14 16 15 12 6		47.4 73.7 73.7 84.2 79.0 63.2 31.6
Other employment No Yes Housework Selling things/running a shop Farming/agricultural Tutoring Tour guide	0 19 10 1 3 5 1		0 100 52.6 5.3 15.8 26.3 5.3
Continuous variables	Mean	Standard deviation	Range
Age	27.9	5.2	21–39
Years at current school	4.5	4.2	1–17
Years teaching	4.9	5.1	1–17

*N* = 21 teachers attending a 10-day training course. Two teachers did not participate further in the study due to changes in employment.

^a^Scheduled caste and scheduled tribe are standard terms used in Indian demographic surveys for officially recognised groups of historically disadvantaged peoples by the Government of India and State of West Bengal. A response of ‘yes’ indicates membership in a historically disadvantaged group.

^b^Sum is greater than 100% due to individuals teaching multiple grade levels.

**Table 2.** Student demographics (*n* = 272)

Categorical variables	Total *n* (%)	Not nominated *n* (%)	Nominated *n* (%)	*P*-value^a^
Number of students	272	215 (79.1)	36 (13.2)	
Sex Female Male	135 (49.6) 137 (50.4)	114 (53.0) 101 (46.9)	15 (41.7) 21 (58.3)	0.21
Grade Class I (1st grade) Class II (2nd grade) Class III (3rd grade) Class IV (4th grade	67 (24.6) 70 (25.7) 66 (24.3) 69 (25.4)	52 (24.2) 57 (26.5) 53 (24.7) 53 (25.7)	10 (27.8) 8 (22.2) 8 (22.2) 10 (27.8)	
Age-grade mismatch^b^ 2 years advanced 1 year advanced Appropriate 1 year delayed 2 years delayed 3+ years delayed	1 (0.4) 16 (5.8) 243 (89.3) 5 (1.8) 6 (1.6) 1 (0.4)	1 (0.5) 14 (6.5) 4 (1.9) 0 (0) 1 (0.5)	0 (0) 0 (0) 1 (2.8) 5 (13.9) 0 (0)	<0.0001**
Mother’s education Some primary Primary Secondary Higher secondary Undergraduate or higher	47 (17.3) 15 (5.5) 172 (63.2) 17 (6.3) 12 (4.4)	39 (18.7) 12 (5.7) 132 (63.2) 15 (7.2) 11 (5.3)	8 (22.9) 3 (8.6) 23 (65.7) 1 (2.9) 0 (0)	0.49
Father’s education Some primary Primary Secondary Higher secondary Undergraduate or higher	37 (13.6) 11 (4.0) 163 (59.9) 36 (13.2) 13 (4.8)	31 (15.0) 8 (3.9) 124 (59.9) 32 (15.5) 12 (5.8)	3 (9.1) 2 (6.1) 25 (75.8) 3 (9.1) 0 (0)	0.30
Scheduled caste or tribe^c^ Yes No	91 (33.5) 181 (66.5)	71 (33.0) 144 (67.0)	11 (30.6) 25 (69.4)	0.77
Language Nepali Bengali English Hindi Other Monthly income category (USD)^d^ 0–99 (%) 100–199 (%) 200–299 (%) >299 (%)	268 (98.5) 1 (0.4) 249 (91.5) 13 (4.8) 0 (0) 151 (55.5) 68 (25.0) 23 (8.5) 27 (9.9)	213 (99.1) 1 (0.5) 196 (91.2) 12 (5.6) 0 (0) 122 (57.3) 55 (25.8) 19 (8.9) 17 (8.0)	36 (100) 0 (0) 36 (100) 0 (0) 0 (0) 18 (51.4) 9 (25.7) 3 (8.6) 5 (14.3)	0.67
Continuous variables	Total mean [range]	Not nominated mean [range]	Nominated mean [range]	*P*-value^a^
Age	8.2 [5–13]	8.1 [5–13]	8.9 [6–13]	0.0062**
Household size^e^ Older generation Same generation Monthly income (USD)^d^	4.4 [1–9] 2.7 [0–8] 0.7 [0–6] 133.27 [7.7–769.2]	4.4 [1–9] 2.7 [0–8] 0.7 [0–6] 125.5 [7.7–769.2]	4.4 [2–7] 2.6 [1–4] 0.8 [0–2] 151.32 [19.2–769.2]	0.81 0.52 0.63 0.34

Students that were nominated as alternates (*n* = 21), but not nominated for intervention, are not included in the nominated or the not-nominated groups.

^a^Calculated using the independent sample t-test for continuous variables, Pearson χ^2^ for categorical variables, or Spearman’s rank correlation for ordinal variables.

^b^Grade outside the expected for age assuming first grade was begun at age 5 or 6 and student advanced 1 grade level each year. Grade-age-mismatch was applied to students that were either young for their grade (advanced) and had a lower mismatch value or old for their grade (delayed) and had a higher age-grade mismatch value.

^c^Scheduled caste and scheduled tribe are standard terms used in Indian demographic surveys for officially recognised groups of historically disadvantaged peoples by the Government of India and State of West Bengal.

^d^Monthly income as reported by family in 2018. Presented in USD (1 USD = 65 INR).

^e^Total number in household including student.

*P < 0.05.

**P < 0.01.

**Figure 1.**
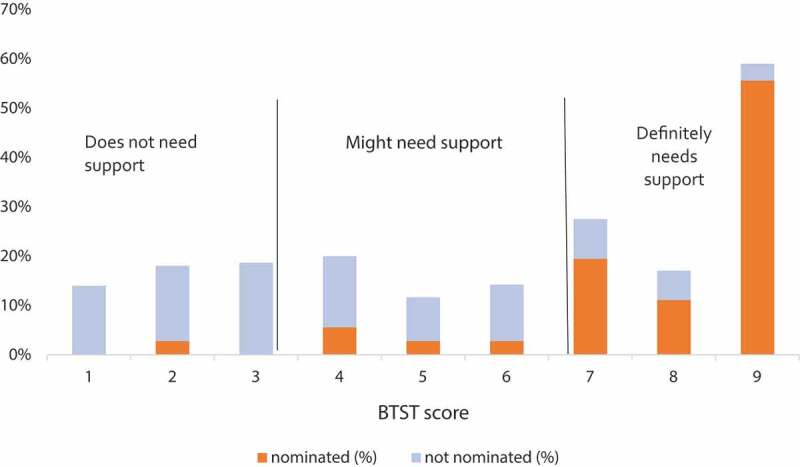
Percent of nominated and not nominated children by BTST score

**Figure 2.**
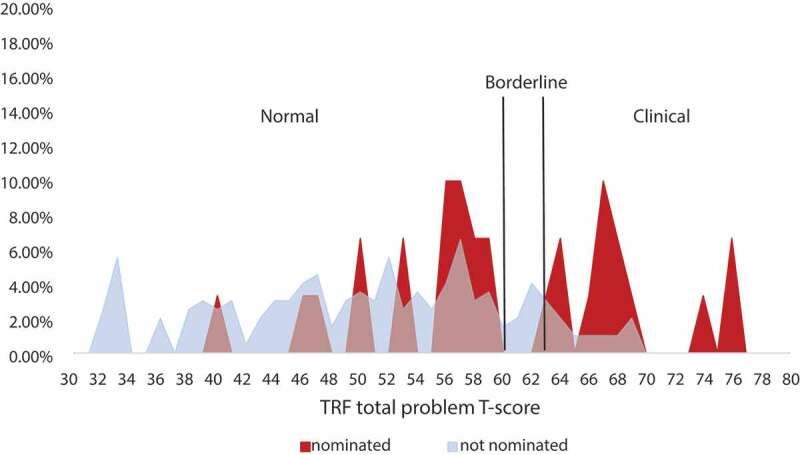
Percent of nominated and not nominated children by TRF total problem T-score

The sensitivity of teacher nomination status to receive the mental health intervention (not-nominated versus nominated as aided by the BTST) by having a clinical or borderline Total Problem or subdomain TRF score, depending on the subdomain, ranged from 0.03 to 0.72, while specificity ranged from 0.63 to 0.97 (Supplemental Table 2). For students having any positive TRF score, sensitivity was 0.72 and specificity was 0.62. Of note, of the 236 children not nominated, 87 children had at least 1 positive TRF subdomain score, 66 (75.9%) of whom had a positive BTST score, defined as having a score indicating that the teacher thought the child ‘might need support’ or ‘definitely needs support’. Figure 1 presents the distribution of the percentage of students nominated and not nominated by their BTST score. Figure 2 displays the distribution of the percentage of students nominated and not nominated by their TRF Total Problem score.

**Figure 3.**
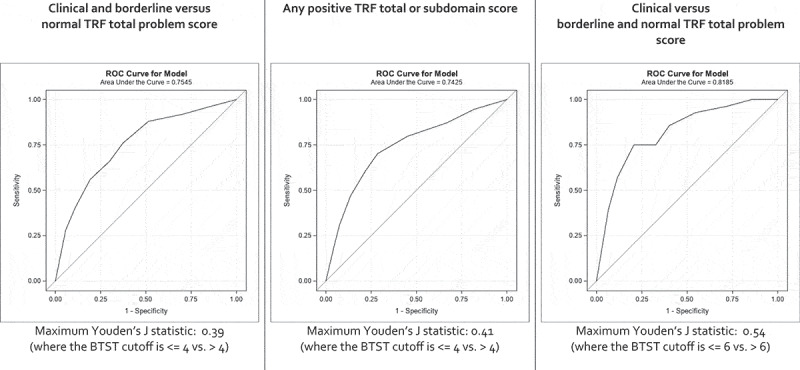
ROC curves for the BTST as compared with the TRF total problem score and any positive TRF score

The BTST overall ranking was statistically significantly weakly correlated with the TRF Total Problem score (*ρ* = 0.34) (Table 3). BTST subdomain scores (yes/no for anxious, disagreeable, and withdrawn) were statistically significantly weakly correlated with both TRF syndrome subdomain scores and TRF DSM-oriented subdomain scores (dichotomized into ‘normal’ and ‘borderline or clinical’), all at the p < 0.001 level (Table 3). BTST overall diagnostic accuracy was moderate, with AUCs ranging from 0.7425 to 0.8185 (Figure 3). The BTST’s maximum differentiating ability was in the low to moderate range, with maximum Youden’s J statistics ranging from 0.39 to 0.54 (Figure 3, Supplemental Table 3). The optimal BTST cutoff was >4 for children with ‘clinical’ or ‘borderline’ struggles, with a maximum Youden’s J of 0.39 when TRF Total Problem score was the standard and 0.41 when any positive TRF total or subdomain score was the standard (Figure 3, Supplemental Table 3). The optimal BTST cutoff level was >6 when comparing children with a clinical TRF Total Problem score, with a maximum Youden’s J of 0.54 (Figure 3, Supplemental Table 3).

**Table 3.** Comparison of BTST and TRF total and subdomain scores

BTST	TRF	Spearman’s correlation coefficient
Overall ranking (fine vs. borderline or needs attention)	Total problem score (normal vs. borderline or clinical)	0.34^
*BTST categories*	*TRF syndrome* *subdomains*	
Anxious	Anxious	0.25^
Disagreeable	Disagreeable	0.23^^
Withdrawn	Withdrawn	0.26^
*BTST categories*	*TRF DSM-oriented* *subdomains*	
Anxious	Anxious	0.33^
Disagreeable	Disagreeable	0.24^
Withdrawn	Withdrawn	0.24^

^*P* < 0.0001.

^^*P* = 0.0002.

The multivariable analysis showed that younger age (*β* = −0.1174, standard error (SE) 0.0167), lower level of education (*β* = −0.0727, SE 0.0129), less formal training in education (*β* = −0.5017, SE 0.1114), and more years of experience (*β* = 0.1345, SE 0.0172) were each significantly associated with higher teacher accuracy (p < 0.001) (Supplemental Table 4). These results suggest that younger teachers who went straight into teaching instead of obtaining more education but had some classroom experience were likely to have more alignment between the BTST and TRF scores they gave each child.

Accounting for individual teachers assessing multiple students and the associated variation in scores at the level of the individual teacher, the multilevel, multivariable analysis showed that less experience (*β* = −0.19, p < 0.04) and less formal training (*β* = −2.64, p < 0.003) were each significantly associated with higher BTST scores at the level of p < 0.05, explaining part of the BTST score variance (Supplemental Table 5). TRF scores were also found to significantly explain part of the BTST score variance (*β* = 0.19, p < 0.0001).

In multivariable regression analyses of student demographic factors associated with nomination, only age and age-grade mismatch were significantly associated, suggesting that the older a child is for their grade (larger delay), the more likely they are to be nominated (Table 2).

## Discussion

This study contributes to the growing literature exploring the role teachers can play in the mental health care of school-aged students in LMICs. To our knowledge, our study documents the first successful application in an LMIC of in-service teacher nomination aided by a decision support tool for primary school children with any mental health symptom for mental health services.

Trained teachers moderately correctly nominated school-aged students with any borderline or clinical TRF Total Problem or sub-score for mental health services without knowing students’ TRF scores, evidenced by a sensitivity of 0.72 and a specificity of 0.62, as hypothesized. This finding supports that trained teachers in an LMIC can recognize as concerning the often-overlooked mental health symptoms of school-aged children. Comparable literature using the same statistical methods and comparing teacher nomination to teacher gold standard forms is sparse [8,15,16,18]. However, the sensitivity is in line with one HIC study where teacher simple nomination had a sensitivity of 0.679 compared to a teacher-completed gold standard measurement (TRF); the study’s specificity, 0.796, was higher than reported here [10].

As the sensitivities and specificities in Supplemental Table 2 may reflect both the teachers’ judgment and the validity of the BTST tool, we compared the performance of the BTST to the TRF, the gold standard. Based on our results, the performance and diagnostic accuracy of the BTST are not sufficient to justify its use for child nomination in isolation (Figure 3, Supplemental Table 3). Coupling it with a process that includes teachers’ judgment, however, resulted in moderate teacher accuracy. Thus, in this study, the ability of trained teachers to accurately nominate children appears to lie predominantly with their judgment, and not in their use of the BTST. This result is similar to HIC teacher simple and in-service nomination studies in which teachers’ judgment and/or training drive accurate nominations [8,10,14–18].

Teacher nomination may have been more accurate than calculated given the use of the TRF (i.e. sensitivity and specificity may have been higher). Previous studies in India using the TRF have indicated that ‘clinical’ and ‘borderline’ cutoff scores may be too high for the Indian setting [43,44]. Notably, a majority of children nominated from the ‘normal range’ had T-scores close to the borderline cut-off (Figure 2). Further, sensitivity and specificity may have been higher had teachers not been restricted to two nominations. Of the students not nominated but who had at least 1 positive TRF subdomain score (n = 87), 75.9% (n = 66) also had a positive BTST score. In light of teacher nomination accuracy being based on their judgment rather than the BTST, this finding suggests that teachers may have nominated more students had a pragmatic cap not existed (Figure 1). Local prevalence rates of 33.3% in a nearby rural region of West Bengal support that more students may have needed mental health services than the 13% pragmatically nominated [1,2].

Younger teachers with some teaching experience and less education more accurately nominated children for mental health services (Supplemental Table 4). Accounting for individual teachers scoring multiple students in the multilevel model, formal training and experience explained part of the variance in BTST scores, having an inverse relationship (Supplemental Table 5). These findings are in line with studies in which early career teachers with some experience demonstrate an increased willingness to learn and use new skills through professional development as compared to more experienced and/or more educated colleagues [53–56]. While this profile may appear contradictory, many participant teachers were in this career stage, starting their careers at younger ages and replacing time they could have spent in training with actual experience in the classroom (Table 1). Notably, this profile is consistent with demographics from other LCP research across India [57,58]. These studies link this teacher profile to LCP schools’ ability to recruit teachers with this level of qualification given the modest salaries offered from the low fees collected [57,58]. Given the wide prevalence of this teacher profile, our findings suggest that LCP teachers are broadly well positioned to take on novel mental health tasks and increase access to care for a population particularly in need, LCP school-aged children who have comparatively poorer access to government services [57,58].

The only student demographic categories significantly associated with increased nomination were Age and Age-Grade Mismatch, which captured students delayed 1–3+ years in school. Clinically, Age-Grade Mismatch, i.e., grade retention, is well known to be associated with mental health struggles [59–62]. Concordantly, children in the nominated group were older than expected for their grade.

### Practice implications

Teacher judgment, rather than decision support tools, appears to underlie the moderate accuracy of teacher nomination in this study. In practice, an emphasis on maximizing teachers’ inherent capabilities, and not on tool creation, will likely be key to accurate teacher nomination in other settings. While a simple tool may be created to correctly map teacher impressions to categories of child mental health struggles, such a measure may take years to develop and robustly validate across the numerous LMIC contexts immediately in need of increased care access [63]. A focus instead on teacher trainings or other capability-enhancing measures could enable the swift use of teacher nomination if evidence grows to support this method.

In light of the severe care gap for children in LMICs, moderate teacher nomination accuracy will allow for at least some of those in need of support to receive it, largely considered a step in the right direction, however imperfect [22,64,65]. Moreover, teachers already possess a keen sense of when a student may be struggling and tend to pick ‘false positives’ who are more likely to be experiencing at least borderline struggles [14,16]. False-negative wellness, by contrast, may lead to potentially dire outcomes for children with unmet mental health needs [64]. Further training on often overlooked mental health concerns (such as depression), increased supervision of teachers, or creative processes such as two teachers conferring on identification may improve false-negative rates. However, lower false-negative rates will need to be balanced with the increased need for resources or teacher time.

Teacher identification is but the first step in increasing access to care. For communities with poor care access, a form of permanent care infrastructure still remains essential to adequately address child mental health needs, necessitating creative solutions [64]. In India, the Government has paid for accredited social health activists (ASHAs), local women who serve as health educators and promoters in their rural communities, and evidence is building in their ability to improve adult mental health outcomes [66]. ASHAs, and community health workers globally, may be a resource for frontline care of common child mental disorders. Other alternatives include parents and families, who have been shown to improve child quality of life through specific interventions [67–69]. Finally, teachers, such as those in this study, may themselves be capable of delivering task-shifted mental health care to their students; they possess expertise in child development and may be able to incorporate therapeutic techniques and interactions throughout instructional time [15].

### Limitations

We obtained data from the real-world implementation of a program. Teachers were limited to two nominations per class, as previously discussed. The TRF may not accurately indicate mental health need in this context, as described above. Teacher nomination, BTST, and TRF were all pragmatically completed by the same teacher for each student, leading to the same potential biases across measures. However, as the BTST was designed to be a short assessment in line with the TRF, a correlation between the two measures was an aim. Moreover, we accounted for this potential bias in our multilevel model. With resource constraints, we were unable to enroll a control group or test enrolled teachers pre-training to examine whether untrained teachers could identify students with mental health struggles accurately. Further, teachers were intensely supported by study staff. This level of guidance may not be feasible at scale without government adoption. Finally, enrollment rates of children with mental health struggles in primary school are not well documented despite anecdotal sentiments that these children are less likely to regularly attend or enroll in school [12]. To understand the potential reach of this referral method, further studies are warranted to quantify enrollment rates of children with mental health struggles and explore why they may not be regularly attending school.

## Conclusions

The results of this study support that, with in-service training and decision support, teachers in an LMIC can nominate school-aged children for mental health services with moderate accuracy. Much of the accuracy lies in teacher judgment after being trained and not with the decision support tool. The findings suggest that the human resources needed to take the first step to close the care gap, child identification and nomination, may already exist in the teacher workforce. Darjeeling is representative of rural regions in LMICs, with lower-paying jobs relative to urban regions of India and with majority minority or marginalized populations, including higher local rates of scheduled caste/scheduled tribe [38]. Such demographics support that teacher nomination of school-aged children is potentially applicable to many LMIC communities, including those with lower literacy rates and resource levels.

Repeat validation of the results in a larger sample size is warranted prior to systematically having teachers identify students in need of mental health support. Additional research is required to assess LMIC teacher capability of identifying children’s mental health struggles without training or support, establish cost-effectiveness, and identify key aspects required for sustainability, including compensation. Findings from the suggested research may support the wider leveraging of teachers as identifiers of children in need of mental health support. This research is an early step towards addressing key research priorities such as in the National Institute of Mental Health ‘Grand Challenges in Global Mental Health Initiative’ and increasing access to children’s mental health care globally [1].

## Supplementary Material

Supplemental Material

## Data Availability

Due to the connectedness of the Darjeeling community and the relatively small sample size of children nominated for mental health intervention, participants of this study did not agree for their data to be shared publicly. Accordingly, supporting data is not available.

## Paper context

To begin to bridge the LMIC child mental health care gap, teachers may be able to nominate students for care. LMIC teacher nomination of school-aged students with any mental health symptom for care has not been studied. Nineteen teachers evaluated 272 students in India. With training and a decision support tool, teachers moderately accurately nominated school-aged children for mental health services. Sustainable methods to engage teachers in student nomination for mental health care are warranted.
