# Supplementary material for: Teacher Nomination of School-aged Children for Mental Health Services in a Low and Middle Income Country
Source: Glob Health Action. 2021 Feb 16;14(1):1861921. doi: 10.1080/16549716.2020.1861921 (PMC7894443; doi:10.1080/16549716.2020.1861921)
Supplement: Supplemental Material [file ZGHA_A_1861921_SM6569.docx]

Figure 1

*Behavior Type and Severity Scale (BTST)*

Teacher Study ID: __________

Class (please circle): I (1) II (2) III (3) IV (4)

*Note: please use only one form per class level.*

Use the behavior rating scale below to classify the behavior of each of your students; record this number in the column labelled “Behavior Rating Scale Number”. Rate each student on a scale of 1 to 9 in terms of their behavior, where 1 is the healthiest behavior and 9 is the most impaired. Students you rank 1-3 are generally students who do not need extra support. Students you rank 4-6 might benefit from support but may also not need it to do well. Students you rank 7-9 definitely need support to do well.

Then, only if appropriate, state whether you believe the student has anxious, disagreeable, or withdrawn behavior in the column “Type of Behavior”. Only record this for students who exhibit these types of behaviors. Some students will not exhibit these types of behaviors and thus will not need for you to state this. If you feel the student has more than 1 type of behavior, rank the behaviors from 1-3, where 1 is the most common behavior for the student.

Recall that anxious behavior is based in an overwhelming and abnormal sense of apprehension, worry, or nervousness. Disagreeable behavior is rooted in needing a sense of control and/or having difficulty with being flexible. Withdrawn behavior comes from not being able to participate in one’s everyday activities.

1

2

3

4

5

6

7

8

9

Healthy

Impaired

**Behavior Rating Scale**

**Does not need support**

**Might need support**

**Definitely needs support**

| **Student Name** | **Behavior Rating Scale Number** | **Type of Behavior** |
| --- | --- | --- |
|  |  |  |
|  |  |  |

Figure 2

*Adapted Logic Model*


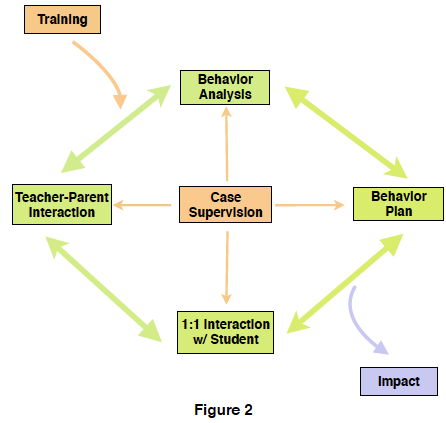


Figure 3

*Timeline of Teacher Training, Data Collection, and Student Nomination Process*

Figure 4

*10-day Training Curriculum Outline*

10-day training focused on identifying students with mental health concerns, completing behavior analyses, creating and adhering to a behavior plan and delivering CBPT

- **Orientation to the Training and Intervention** (Day 1, ½ day)
  - **Pre-training summative assessment**
- **Module 1: Understanding Behavior** (Day 2)
  - **Introduction to Behavior**
  - **Behavior has specific functions or motives and can have more than one function**
  - **Behavior can be productive or disruptive**
  - **Behavior is triggered, leading to patterns of behavior**
  - **Poor behavior may indicate a missing skill**
  - **Poor behavior is often atypical behavior**
  - **Building relationships: the key to behavior change**
  - **Living Example**
- **Module 1.5: Approaching Your Students and Community** (Day 3)
  - **Why do the details of communication matter?**
  - **Why are behavioral issues so sensitive?**
  - **Communicating with students**
  - **Communicating with families**
  - **Communicating with colleagues**
  - **The power of relationships**
  - **Self-awareness: recognizing your own biases while supporting the student**
- **Module 2: The 4Cs Plan** (Day 4 and 5)
  - **What are behavior plans?**
  - **What is the 4Cs plan?**
  - **4Cs: Cause**
  - **4Cs: Change**
  - **Teaching new skills**
    - **Cognitive Behavioral Play Therapy (CBPT)**
  - **4Cs: Connect**
  - **4Cs: Cultivate**
  - **Choosing the Right 4Cs Plan**
- **Module 3: Working with Nervous Children** (Day 6)
  - **Introduction to Anxiety**
  - **4Cs: Cause for anxious students**
  - **4Cs: Change for anxious students**
    - **CBPT**
  - **4Cs: Connect for anxious students**
  - **4Cs: Cultivate for anxious students**
  - **Creating a 4Cs plan for a student with anxiety**
- **Module 4: Working with Disagreeable Children** (Day 7)
  - **What does it mean to be “disagreeable”?**
  - **4Cs: Cause for students with disagreeable behavior**
  - **4Cs: Change for disagreeable students**
    - **CBPT**
  - **4Cs: Connect for disagreeable students**
  - **4Cs: Cultivate for disagreeable students**
  - **Creating a 4Cs plan for a disagreeable student**
- **Module 5: Working with Withdrawn Children** (Day 8)
  - **What does it mean to be “withdrawn”?**
  - **4Cs: Cause for students with withdrawn behavior**
  - **4Cs: Change for withdrawn students**
    - **CBPT**
  - **4Cs: Connect for withdrawn students**
  - **4Cs: Cultivate for withdrawn students**
  - **Creating a 4Cs plan for a withdrawn student**
- **Review of Study Protocols** (Day 9)
  - **Child Abuse**
  - **Suicidal Ideation or Attempt**
- **Conclusion** (Day 10, ½ day)
  - **Feedback from participants about training**
  - **Post-training summative assessment**

Table 1

*TRF Categories Mapped to BTST Category*

| BTST | TRF |
| --- | --- |
| Overall Ranking | Total Problem Score |
| *BTST Category* | *TRF Syndrome Scales* |
| Anxious | Anxious/depressed, Somatic complaints, Social problems, Thought problems, Attention problems, Rule-breaking behavior, Aggressive behavior |
| Disagreeable | Anxious/depressed, Social Problems, Thought problems, Attention problems, Rule-breaking behavior, Aggressive behavior |
| Withdrawn | Withdrawn/depressed, Social problems, Attention problems |
| *BTST Categories* | *TRF DSM-oriented*  *Scales* |
| Anxious | Anxiety problems, Somatic problems, Attention deficit, Oppositional Defiant problems, Conduct problems |
| Disagreeable | Anxiety problems, Attention deficit, Oppositional Defiant problems, Conduct problems |
| Withdrawn | Depressive problems, Attention deficit |

Table 2

*Baseline Student Mental Health Profile: Sensitivity and Specificity of Nomination for Mental Health Intervention to Children’s TRF Overall and Subdomain Scores*

| Symptom Score | Total  N=272 | Nominated  N=36 | Not Nominated  N=236 | Sensitivity | Specificity |
| --- | --- | --- | --- | --- | --- |
| Total Problem Score*  Borderline or Clinical  Normal^a^ | 59  214 | 17  19 | 42  194 | \| 0.47 \| \| --- \| \|  \| | 0.82 |
| Internalizing Problems*  Borderline or Clinical  Normal^a^ | 80  193 | 19  17 | 61  175 | \| 0.53 \| \| --- \| \|  \| | 0.74 |
| Externalizing Problems  Borderline or Clinical  Normal^a^ | 47  226 | 11  25 | 36  200 | \| 0.31 \| \| --- \| \|  \| | 0.85 |
| Any Symptom*  Borderline or Clinical  Normal^b^ | 91  181 | 25  11 | 66  170 | \| 0.69 \| \| --- \| \|  \| | 0.72 |
| Any DSM Diagnosis*  Borderline or Clinical  Normal^b^ | 81  190 | 21  14 | 60  176 | \| 0.58 \| \| --- \| \|  \| | 0.75 |
| Any positive score*  Borderline or Clinical  Normal^ab^ | 113  159 | 26  10 | 87  149 | \| 0.72 \| \| --- \| \|  \| | 0.63 |
| Withdrawn/ Depressed*  Borderline or Clinical  Normal^b^ | 47  226 | 16  20 | 31  205 | \| 0.03 \| \| --- \| \|  \| | 0.87 |
| Social Problems*  Borderline or Clinical  Normal^b^ | 31  242 | 11  25 | 20  216 | \| 0.31 \| \| --- \| \|  \| | 0.92 |
| Inattention Problems*  Borderline or Clinical  Normal^b^ | 27  246 | 11  25 | 16  220 | \| 0.28 \| \| --- \| \|  \| | 0.95 |
| Hyperactive-Impulsive  Borderline or Clinical  Normal^b^ | 12  261 | 4  32 | 8  228 | \| 0.11 \| \| --- \| \|  \| | 0.97 |
| Depressive Problems*  Borderline or Clinical  Normal^b^ | 50  223 | 14  22 | 36  200 | \| 0.39 \| \| --- \| \|  \| | 0.85 |
| Anxiety  Borderline or Clinical  Normal^b^ | 34  239 | 9  27 | 25  211 | \| 0.25 \| \| --- \| \|  \| | 0.89 |
| Somatic Complaints  Borderline or Clinical  Normal^b^ | 22  251 | 6  30 | 16  220 | \| 0.17 \| \| --- \| \|  \| | 0.93 |
| Inattentive-Hyperactive  Borderline or Clinical  Normal^b^ | 17  256 | 6  30 | 11  225 | \| 0.17 \| \| --- \| \|  \| | 0.95 |
| Any Anxious Category*  Borderline or Clinical  Normal^b^ | 75  198 | 19  17 | 56  180 | \| 0.53 \| \| --- \| \|  \| | 0.76 |
| Any Withdrawn Category*  Borderline or Clinical  Normal^b^ | 65  208 | 21  15 | 44  192 | \| 0.58 \| \| --- \| \|  \| | 0.81 |
| Any Disagreeable Category*  Borderline or Clinical  Normal^b^ | 64  209 | 18  18 | 46  190 | \| 0.50 \| \| --- \| \|  \| | 0.81 |
| Any DSM Anxious Diagnosis*  Borderline or Clinical  Normal^b^ | 69  204 | 19  17 | 50  186 | \| 0.53 \| \| --- \| \|  \| | 0.79 |
| Any DSM Withdrawn Diagnosis*  Borderline or Clinical  Normal^b^ | 60  213 | 17  19 | 43  193 | \| 0.47 \| \| --- \| \|  \| | 0.82 |
| Any DSM Disagreeable Diagnosis*  Borderline or Clinical  Normal^b^ | 56  217 | 16  20 | 40  196 | \| 0.44 \| \| --- \| \|  \| | 0.83 |
| Oppositional Defiant Problems*  Borderline or Clinical  Normal^b^ | 12  261 | 6  30 | 6  230 | \| 0.17 \| \| --- \| \|  \| | 0.97 |
| Conduct Problems*  Borderline or Clinical  Normal^b^ | 20  253 | 8  28 | 12  224 | \| 0.22 \| \| --- \| \|  \| | 0.95 |
| Inattention Problems*  Borderline or Clinical  Normal^b^ | 21  252 | 10  26 | 11  225 | \| 0.28 \| \| --- \| \|  \| | 0.95 |
| Hyperactive/ Impulsive  Borderline or Clinical  Normal^b^ | 13  260 | 4  32 | 9  227 | \| 0.11 \| \| --- \| \|  \| | 0.96 |
| Slow Cognitive Performance*  Borderline or Clinical  Normal^b^ | 32  271 | 10  26 | 22  214 | \| 0.28 \| \| --- \| \|  \| | 0.91 |
| Obsessive Compulsive*  Borderline or Clinical  Normal^b^ | 25  248 | 11  25 | 14  222 | \| 0.31 \| \| --- \| \|  \| | 0.94 |
| Stress Related Problems*  Borderline or Clinical  Normal^b^ | 41  232 | 17  19 | 24  212 | \| 0.47 \| \| --- \| \|  \| | 0.90 |

Dx = Diagnosis.

^a^ Scores lower than the 83^rd^ percentile were considered normal for the internal, external and total problem scales.

^b^ Scores lower than the 93^rd^ percentile were considered normal for syndrome and DSM oriented scales.

* chi-square p < 0.0019 (Bonferroni corrected alpha: 0.05/27)

Table 3

*Sensitivities, specificities, and Youden’s Js for BTST cutoffs*

| **Clinical and borderline vs. normal TRF Total Problem score** | | | | |
| --- | --- | --- | --- | --- |
| BTST cutoff | Sensitivity | 1-Specificity | Specificity | Youden's J |
| 1 | 0.96 | 0.85 | 0.15 | 0.11 |
| 2 | 0.92 | 0.71 | 0.29 | 0.21 |
| 3 | 0.88 | 0.51 | 0.49 | 0.37 |
| **4** | **0.76** | **0.37** | **0.63** | **0.39** |
| 5 | 0.66 | 0.3 | 0.7 | 0.36 |
| 6 | 0.56 | 0.19 | 0.81 | 0.37 |
| 7 | 0.4 | 0.11 | 0.89 | 0.29 |
| 8 | 0.28 | 0.06 | 0.94 | 0.22 |
|  |  |  |  |  |
| **Clinical vs. borderline and normal TRF Total Problem score** | | | | |
| BTST cutoff | Sensitivity | 1-Specificity | Specificity | Youden's J |
| 1 | 1 | 0.85 | 0.15 | 0.15 |
| 2 | 0.96 | 0.72 | 0.28 | 0.24 |
| 3 | 0.93 | 0.55 | 0.45 | 0.38 |
| 4 | 0.86 | 0.4 | 0.6 | 0.46 |
| 5 | 0.75 | 0.33 | 0.67 | 0.42 |
| **6** | **0.75** | **0.21** | **0.79** | **0.54** |
| 7 | 0.57 | 0.12 | 0.88 | 0.45 |
| 8 | 0.39 | 0.07 | 0.93 | 0.32 |
|  |  |  |  |  |
| **Any Positive TRF total or subdomain score** | | | | |
| BTST cutoff | Sensitivity | 1-Specificity | Specificity | Youden's J |
| 1 | 0.95 | 0.82 | 0.18 | 0.13 |
| 2 | 0.87 | 0.67 | 0.33 | 0.2 |
| 3 | 0.8 | 0.45 | 0.55 | 0.35 |
| **4** | **0.7** | **0.29** | **0.71** | **0.41** |
| 5 | 0.61 | 0.22 | 0.78 | 0.39 |
| 6 | 0.47 | 0.14 | 0.86 | 0.33 |
| 7 | 0.31 | 0.08 | 0.92 | 0.23 |
| 8 | 0.19 | 0.05 | 0.95 | 0.14 |

*Bolded values indicate maximum Youden’s J per table*

Table 4

*Multivariable association of teacher demographic characteristics with “rho” scores reflecting the correlation of the BTST and the TRF.*

| Demographic factor | Beta estimate | Standard Error |
| --- | --- | --- |
| Age * | -0.1174 | 0.0167 |
| Experience * | 0.1345 | 0.0172 |
| Education * | -0.0727 | 0.0129 |
| Formal Training * | -0.5017 | 0.1114 |

*p<0.001

Table 5

*Multilevel multivariable analysis of the association of BTST (outcome) with teacher demographic characteristics and TRF .*

| Demographic factor | Beta estimate | Standard Error | p-value |
| --- | --- | --- | --- |
| Age | -0.02 | 0.08 | 0.78 |
| Sex | 0.40 | 0.57 | 0.48 |
| Experience* | -0.19 | 0.091 | 0.04 |
| Education | -0.18 | 0.11 | 0.11 |
| Formal Training* | -2.64 | 0.89 | 0.003 |
| TRF score* | 0.19 | 0.02 | <0.0001 |

*p<0.05
